# Parents’ Knowledge, Attitude and Perceptions on Childhood Vaccination in Saudi Arabia: A Systematic Literature Review

**DOI:** 10.3390/vaccines8040750

**Published:** 2020-12-10

**Authors:** Marwa Alabadi, Zakariya Aldawood

**Affiliations:** 1 School of Nursing and Midwifery, Faculty of Health and Medicine, University of Newcastle, Callaghan, NSW 2308, Australia; 2 Primary Health Care Division of Qatif City, General Directorate of Health Affairs in the Eastern Region, Ministry of Health, Qatif 31911, Saudi Arabia; zakariyaa@moh.gov.sa

**Keywords:** vaccine, childhood vaccination, immunisation in Saudi Arabia, parents, children, knowledge, attitude, parental perceptions

## Abstract

(1) Background: The responsibility of vaccinating children lies with their parents. Therefore, parents’ attitudes, knowledge and perceptions towards vaccination are of great importance as it drives their actions for timely and complete immunisation. This systematic literature review was conducted to gain a better understanding of parents’ knowledge, attitudes and perceptions regarding childhood vaccination in Saudi Arabia. (2) Methods: A comprehensive systematic literature review was conducted to identify evidence demonstrating parents’ knowledge, attitudes and perceptions on childhood vaccination in Saudi Arabia. The Preferred Reporting Items for Systematic Reviews and Meta-Analyses (PRISMA) reporting guidelines were used for this review. (3) Results: Nine studies were subsequently included in this systematic review. (4) Conclusions: All the individual reports in the literature do not cover the entire nation of Saudi Arabia, indicating the necessity of more comprehensive investigations so that the government and policymakers can develop versatile fact-based policies for the well-being of future generations.

## 1. Introduction

Before the introduction of routine vaccines, infectious diseases were among the most common causes of mortality in children globally. Administration of a vaccine takes place in the form of oral dosage or injection of killed formulations or live attenuated disease-producing organisms. Following this, an individual develops or produces antibodies for prevention and active immunity development. Immunisation is the process through which vaccines stimulate the development of the immune system in individuals. Among the medical interventions for the prevention of infectious diseases, immunisation is one of the most cost-effective and viable methods. It has been known to prevent several deadly diseases, including meningitis, diphtheria, hepatitis B, measles, mumps, polio, pertussis, rubella, pneumonia, tetanus and rotavirus diarrhea [1].

Vaccination, one of the most powerful weapons against vaccine-preventable infectious diseases, saves millions of lives every year [2]. However, despite the provisions of free health care and readily available vaccines, a significant proportion of children do not receive their childhood vaccinations across the globe, and the situation intensifies in developing countries [3].

Saudi Arabia, a developing country, began its immunisation programme in 1964 and used Bacille Calmette–Guerin (BCG) vaccine to contain tuberculosis (TB) disease. The Expanded Programme on Immunisation (EPI) was later expanded to include poliomyelitis, diphtheria, pertussis, tetanus and measles. The programme has been implemented as an essential and integrated element of Primary Health Care (PHC) since 1984 (see Appendix C). The EPI was introduced to the Saudi population for free under the sponsorship of the Ministry of Health. According to the ministry of health surveillance data, overall vaccine uptake is good in Saudi Arabia and has significantly reduced mortality and morbidity among children from the target diseases [4]. However, similar to most countries around the world, Saudi Arabia faces the challenge of an uneven vaccination uptake among its population. Additionally, inadequate vaccination is a problem that not only puts the children at risk of contracting these vaccine-preventable diseases, but also causes substantial humanitarian and economic burdens in the long run.

The responsibility the vaccinate children’s lies with their parents. Therefore, parents’ attitudes, knowledge and perceptions towards vaccination are of great importance as they drive their actions for timely and complete immunisation. However, previous studies indicate a lack of knowledge and awareness among Saudi parents which contributed to their negative attitude towards childhood immunisations [5]. Similarly, another study conducted to assess parents’ immunisation knowledge found that 20–40% of the respondents had insufficient knowledge on the topic [6].

This study is therefore conducted to gain a better understanding of parents’ knowledge, attitudes and perceptions (KAPs) regarding childhood vaccination. Although childhood vaccination is the major focus of this review, we have also included studies on seasonal influenza vaccination to extend the evidence base of this study.

### Objectives

There are three objectives of this systematic review as follows:

1. To describe the KAPs of parents towards childhood vaccination in Saudi Arabia.
2. To explore the reasons for the delayed and non-vaccination of children.
3. To highlight the evidence gap and make recommendations for the relevant interventions based on the findings of the systematic review.

## 2. Methods

This systematic review was conducted to identify evidence demonstrating parents’ KAPs on childhood vaccination. The Preferred Reporting Items for Systematic Reviews and Meta-Analyses (PRISMA) reporting guidelines were used for this systematic literature review [7]. By using PRISMA guidelines, we aimed to apply a rigorous and transparent methodology, which minimises any bias in the selection of relevant studies and data.

### 2.1. Eligibility Criteria

The past ten years of literature published in the English language was audited so that studies reporting Saudi parents’ knowledge, attitudes and perceptions on childhood vaccination could be identified and included in this systematic review. The detailed eligibility criterion reported below in Table 1 was systematically applied.

### 2.2. Data Sources and Search Strategy

Databases searched for this review included: Medline, Embase, Scopus, Cinhal, PsychINFO, Web of Science, Cochrane library and ProQuest. The ProQuest search encompassed 13 databases, the details of which are mentioned in Table 2.

The search strategy used for the Medline and Embase databases is reported in Appendix A and was developed from search terms relating to KAPs to vaccination. The search was refined by applying filters to limit the studies to the past ten years (September 2010 to September 2020), the English language and humans. The search was not limited by age group of children at this stage. The bibliographies of any relevant studies were also screened to identify additional relevant studies. The search results were downloaded and imported into the reference management software “Endnote” [8].

### 2.3. Study Selection

The studies to be audited were screened by both authors with the abstract screening software “Rayyan” [9] via title and abstracts. Research studies including Saudi parents with immunisation responsibilities for children and reporting KAPs regarding childhood vaccination in English were included as data in our research.

The full texts of all studies that met the inclusion criteria for the title and abstract screening were obtained. Full texts were subsequently screened using the same inclusion criteria as applied during the previous abstract screening step. On this second pass, however, the focus was on identifying studies with the relevant outcomes (KAP studies on vaccinations).

### 2.4. Interpretation and Data Extraction

The relevant data were then extracted into a preagreed Microsoft Excel template. The following data fields were extracted from each study which fulfilled all of our inclusion criteria:

1. Study details: study name, study design, year of publication, study setting, country, recruitment method, number of study centres, inclusion and exclusion criteria and sample size.
2. Parents’ characteristics: age, gender, education level, income, employment status, residence area (rural/urban), number of children under their care and age of the youngest child.
3. Children’s characteristics: age and immunisation status.
4. Data collection instruments and characteristics: name, reference and characteristics.
5. Outcomes: knowledge, attitude, perceptions and reasons for not vaccinating.

### 2.5. Quality Appraisal

All included studies were assessed using the Joanna Briggs institute (JBI) reviewers manual tools for a systematic review of prevalence and incidence studies [10]. The risks of a bias table and graph were prepared using a software called “Review Manager 5” [11]. The results were synthesised narratively to identify common themes and gaps within the data.

## 3. Results

**Figure 1:**
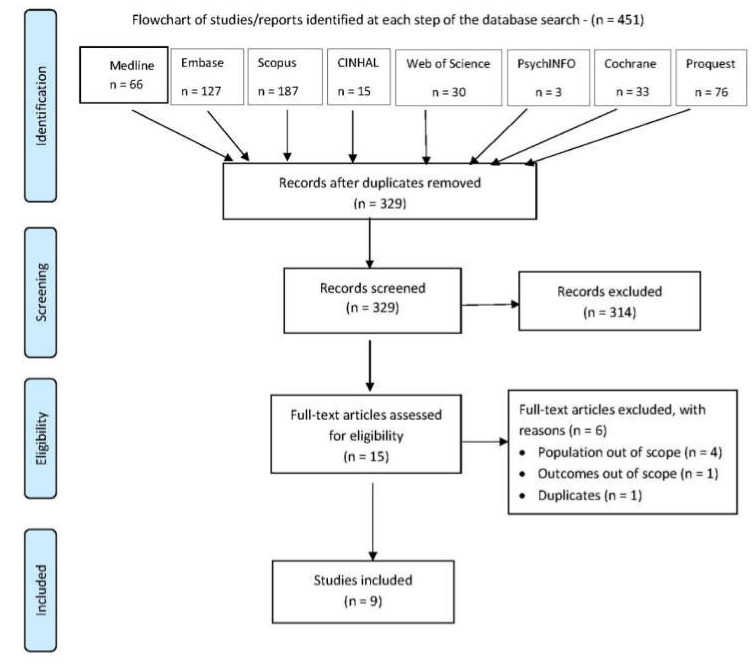
Preferred Reporting Items for Systematic Reviews and Meta-Analyses (PRISMA) flow diagram.

The database search identified 451 citations, of which 122 were duplicates, leaving 329 unique citations to be further screened as shown in Table 2. Fifteen articles were identified as potentially meeting the inclusion criteria and were retrieved as full texts. Of this group, six were excluded as they did not meet the inclusion criteria. The remaining nine studies were subsequently included in this systematic review as shown in Figure 1.

### 3.1. Studies Characteristics

Nine studies included in this systematic review described primary research on the knowledge, attitude and perception of parents on childhood vaccination. Seven of these studies focussed on childhood vaccinations while the remaining two focus on seasonal influenza vaccination. All studies used observational cross-sectional methodologies. All studies were conducted in Saudi Arabia: 5 were conducted in Riyadh, 1 in Jeddah, 1 in Taif, 1 in the Hail region and 1 in the Qasim region. All studies were published in the English language.

The nine studies identified following the database search and screening included a total of reporting 3502 parents as participants. Convenience sampling was the most common method of sampling (n = 6) and only two studies used a random sampling method. The range of children’s age varied across all nine studies. A summary of the nine included studies is detailed in Table 3.

### 3.2. Participants’ Characteristics

All participants in the nine studies were adult parents; however, the range of education levels varied vastly among parents, from illiterate to Ph.D level. Additionally, none of the nine studies used the same or a similar scale to report the education levels of the participants. More than half of the participating parents were females. Women comprised over 90% of the participants in [13]; however, gender data were not available for two of the studies [12,16]. The number of children under the respondents’ care ranged from 1 to over 7, although the majority of the parents had 2 to 4 children.

### 3.3. DCIs Characteristics

There was extensive variation between the data collection instruments (DCIs) used to assess the parents’ KAPs across all nine studies. The number of sections per questionnaire and the number of questions per section varied for all included studies primarily because all of them had used different DCIs. The characteristics of the DCIs used for each of the studies are reported in Table 4.

### 3.4. Quality Assessment

**Figure 2:**
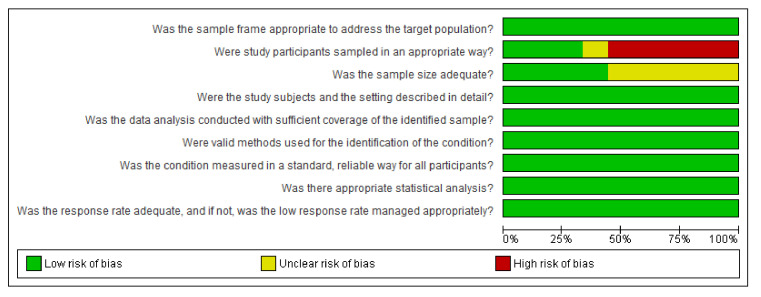
Risk of bias graph.

**Figure A1:**
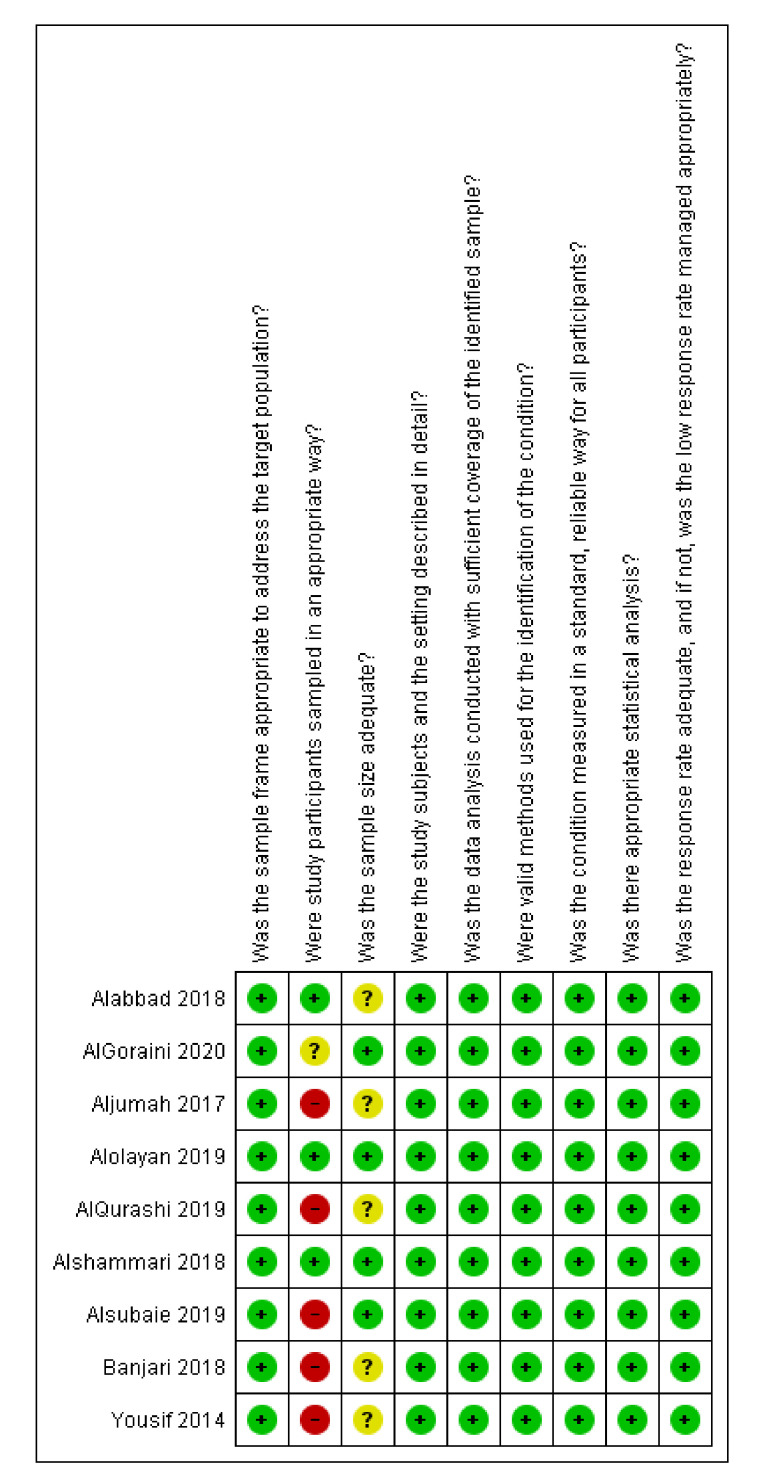
Risk of bias summary. (Green: Low risk of bias, Yellow: Unclear risk of bias, Red: High risk of bias).

The risk of bias assessment for each of the nine studies is summarised in Figure 2 and Figure A1.

The Risk of Bias (ROB) was low in 7 of the 9 items of the quality appraisal tool over the nine studies. However, 6 of 9 studies have a high ROB for sampling methodology as the participants were selected using a convenience sampling method. One of the remaining studies had an unclear ROB and the remaining two studies selected the participants through a random sampling method; hence, they had a low ROB. The authors did not report any required sample size calculations in 5 of the 9 studies included in our investigation; therefore, they had an unclear ROB.

### 3.5. Knowledge of Parents

Five of the nine studies reported the knowledge level of the adult participants. Two of these studies reported a good level of vaccination knowledge [12,15]; the other two studies reported good knowledge on some aspects and poor on others [16,17], while one reported substantial lack of knowledge [18].

AlGoraini et al. [12] reported that the majority of the parents (78%) were aware of the importance of a vaccine’s protective effect on their children’s health. However, the authors found that approximately 15% of participants were hesitant to vaccinate their children. Similar results were seen in the study conducted by Alshammari et al. [15], who found that 60–90% of participants knew the importance of vaccination for their children and about 390 out of 403 parents had their children fully vaccinated (86%).

However, the knowledge level the parents reported varied across Saudi Arabia, as did the depth of information they knew of the vaccination processes and details. For instance, Aljumah et al. [16] reported that knowledge level was high for the vaccines’ protective effect and timing of the first dose in a vaccination schedule (95.2%), and (86.9%), respectively. However, the knowledge levels on dosages and the impact of multiple vaccines on child immunity were 41.6 and 47%, respectively [16]. Similarly, Yousif et al. reported in [17] that 672 out of 731 (92%) parents appreciated the protection afforded by vaccines against infectious diseases and 634 out of 731 (87%) of the parents were mindful of the first dose’s timing. However, Yousif et al. [17] also documented a lack of knowledge among parents on the importance of administering multiple doses (41.6%), administering multiple vaccines on child immunity (37.1%), seasonal influenza vaccination (45.7%) and contraindication to vaccination (39.3%). Furthermore, Alolayan et al. [18] reported an overall poor level of knowledge among 246 out of 399 parents (61.7%).

### 3.6. Attitude of Parents

Five of the nine studies reported the parents’ attitudes on their children’s vaccination rates and levels. Overall, the attitude of parents towards vaccination was positive. Three of these studies focussed on childhood vaccination and two focussed on the influenza vaccine. It was reported in [16] that 87% of parents adhered to the vaccination programme while Alshammari et al. [15] reported that 408 out of 453 parents (90%) encouraged others to get their children vaccinated. In total, 719 out of 731 parents (98.4%) strongly agreed that childhood immunisation was necessary, and 669 (91.5%) believed vaccination is more beneficial than harmful [17].

Alolayan et al. [18] found that 94.7% of participating parents had a positive attitude towards the seasonal influenza vaccine. The women had a more significantly positive attitude than men. Overall, studies reported that a positive attitude was associated with a higher level of education (*p* = 0.02) [18].

### 3.7. Perception/Hesitancy of Parents

In this study, vaccination hesitancy refers to any parental delay in acceptance or refusal of shots despite the availability of vaccine services. This delay could also be influenced by factors such as complacency and convenience. Vaccine hesitancy of childhood was reported in three studies [12,13,20]. Alabbad et al. [20] reported that 51 out of 300 parents were hesitant to give their children the required vaccination. However, Alsubaie et al. [13] reported that 100 out of 500 patents (20%) were reluctant to get their child vaccinated while AlGoraini et al. [12] found that 57 out of 384 participants (14.8%) were hesitant to vaccinate their children. None of the studies that we looked at in detail measured the perceptions of parents.

### 3.8. Sources of Vaccination Information

The main source of vaccination information for parents across the nine studies was a physician or medical staff member. However, people also benefited from awareness campaigns and media reports. Interestingly, parents appeared to be very receptive to friends and family’s recommendations for vaccination [15]. A higher vaccination rate among children was observed by Alsubaie et al. [13], as 34.4% of participants indicated that children in their families had been vaccinated.

### 3.9. Reasons for Low Vaccine Uptake

Seven of the nine studies reported a range of reasons for delayed or missing childhood vaccinations. Banjari et al. [14] reported that 31 out of 142 participants (21.3%) indicated that travelling on vaccination day was the most common reason for the delay, followed by the unavailability of the vaccine in the health care facility (15.5%). Other reported reasons included transport issues and no time to make a trip to the health care centre [14].

A significant proportion of children in Saudi Arabia is partially vaccinating. This was mainly due to a lack of education [16]. Parents of partially vaccinated children believed that their children did not need vaccines for diseases that are no longer prevalent and that too many shots in one visit are not suitable for their children [13,17]. Travelling distance to and from the respective vaccination centres was another reason for missing vaccination doses [15].

The reasons for low vaccine uptake of the influenza vaccine were reported to be different from those reasons given for routine childhood vaccines. Parents believed that their children did not need an influenza vaccine because influenza was a simple health problem and the natural immunity within the children and their communities was better than the immunity afforded by the vaccine. Surprisingly, some parents thought that the influenza vaccine could cause an influenza infection or other adverse effects. Additionally, parents had doubts about the vaccine’s efficacy and believed that their physician did not recommend influenza vaccinations [18,20].

## 4. Discussion

Based on our review of the current literature, the overall knowledge level of parents on childhood vaccination is considered good in Saudi Arabia. However, the knowledge that parents displayed varied greatly across the various categories of information relevant to vaccines. For instance, parents’ knowledge level about the availability of vaccine was high [17], but the effectiveness of multiple vaccines in one visit was low [13]. Additionally, Alqurashi et al. [19] reported that parents with higher education had higher immunisation rates for their children [18]. However, these results are contradictory with the findings of Alsubaie et al. [13] who found that parents with a postgraduate degree tended to be more vaccine-hesitant compared with parents who had a bachelor’s degree or school degree (*p* < 0.001).

Generally, parents’ attitudes towards their children vaccination was positive. However, similar to the knowledge level, the range of responses from the parents showed enormous variation. Surprisingly, parents’ high education levels did not contribute to the high vaccine uptake of their children [20]. Alolayan et al. [18] reported that a more positive attitude towards seasonal influenza vaccination was associated with those parents who worked in a medical field (*p* = 0.02). In contrast, however, Alabbad et al. [20] reportedly found no significant association between education levels of parents and their children receiving the influenza vaccination. 

Although the focus of this study was childhood vaccination, we also included studies that investigated influenza vaccination and included assessments of parents KAP. Additionally, the data from mixed population studies was included in the current study. For instance, Alabbad et al. [20] was a mixed population study conducted on adults, parents and health care workers. We, however, included only the parents’ data from Alabbad et al. [20] for completeness of our systematic review.

### Limitations of Included Studies

Although the authors used pretested and validated DCIs, no two DCIs within the nine studies were identical. Extensive variations within the DCI tools caused major differences in the ways the outcomes could be measured and compared.

Moreover, the majority of the studies have used a convenience sampling methodology to select the participants. Although this may be the only option for selecting the subjects, primarily if the survey was conducted only in one centre, it certainly induces a high ROB for the data generated in each of the research studies. Additionally, more than half of the studies did not report the required sample size. Both of these limitations have generated doubts about the validity and scope of the study’s outcomes and conclusions.

In addition, some of the studies were conducted in the Central Region of Saudi Arabia. They did not include other regions due to limited access to a more significant sample from different areas of the country. Additionally, some other studies included only patients who were admitted at the authors’ tertiary governmental care hospital and did not include others who were not admitted or were admitted elsewhere. Furthermore, most of the included studies took place in a single region and may not be generalizable to other areas of Saudi Arabia.

## 5. Conclusions

The main goal of this study is to contribute to a body of knowledge that can enhance public health practice in general and lead to more effective immunisation programmes in Saudi Arabia. This study is the first systematic literature review conducted to assess and describe parents’ knowledge, attitudes and perceptions towards childhood vaccination in Saudi Arabia. No systematic literature reviews were found within any of the digital libraries included in our methodology section. Furthermore, all the individual reports in the literature do not cover the entire nation of Saudi Arabia, indicating the necessity of more comprehensive investigations so that the government and policymakers can develop versatile fact-based policies and proper educational materials for the well-being of future generations.

## Figures and Tables

**Table 1:** Inclusion and exclusion criteria.

Category	Inclusion Criteria	Exclusion Criteria
Population	Adult parents, carers or guardians (over 18 years) who had at least one child and were responsible for that child’s vaccination. Studies assessing the parents/guardians/carers knowledge, attitude, practices and perceptions regarding children’s vaccination.	Studies investigating parents’ attitudes, knowledge and perceptions on interventions other than childhood vaccination. Studies assessing efficacy or safety of vaccines.
Outcomes	The outcomes consisted of at least one of the following: Knowledge, Attitudes, Perceptions (KAPs).	
Study Design	Randomised controlled trials. Non-randomised studies. Cohort studies. Case-control studies. Cross-sectional studies. Systematic Literature Reviews (SLRs)/Network Meta-Analyses (NMAs) ^1^	Letters to the editor. Narrative reviews. Editorials. Expert opinions. Case studies.
Geographical location	Kingdom of Saudi Arabia (studies conducted on Saudi population).	All other countries
Year of Publication	Sep 2010 to Sep 2020	Studies before Sep 2010
Language	English	Non-English
Filters Applied: Human		

^1^ Relevant Systematic Literature Reviews (SLRs) and Network Meta-Analyses (NMAs) will be included and references will be reviewed to identify any additional relevant publications.

**Table 2:** Total number of studies identified from database searches.

Source	Number of Hits	Total after De-Duplication
Medline	66	329
Embase	127	
CENTRAL	33	
Scopus	187	
CINHAL	15	
PsychINFO	3	
Web of Science	30	
ProQuest	76	

**Table 3:** Summary of included studies.

R	Aim of the Study	Participants (n)	Vaccine Focus	Kid’s Age	Conclusions
[12]	To assess the magnitude of hesitancy of parents towards vaccines and to determine the reasons parents either partially vaccinate and/or do not vaccinate children in Riyadh, Saudi Arabia	384	National Childhood Immunisation Program	<14 years	Confidence towards vaccinations is good among parents in Riyadh, Saudi Arabia. Only a minority of the parents showed hesitancy
[13]	To assess the prevalence of vaccine hesitancy among Saudi parents along with its determinants	500	National Childhood Immunisation Program	2 months–7 years	Vaccine hesitancy is a major concern among parents in the Kingdom of Saudi Arabia (KSA) suggesting risk to the currently reported high vaccination level. Vaccination status of children cannot be used as the main indicator to assess vaccine hesitancy, as it does not consider parents who have significant concerns towards vaccines
[14]	To assess vaccination timeliness, risk factors associated with delays and the reasons for delayed vaccinations among children below the age of 3 years in Jeddah, Saudi Arabia.	351	National Childhood Immunisation Program	<3 years	Adherence to vaccination is fairly common in this part of the country. However, vaccination delays are still present and should be addressed to improve health care
[15]	To assess perceptions of and attitudes towards routine childhood immunisation among Saudi parents	467	National Childhood Immunisation Program	<5 years	Confidence in and acceptance of childhood vaccinations and perceptions of vaccine-related health benefits were quite good among Saudi parents. Parents also appeared to have easy access to diverse sources of vaccine-related information and education
[16]	To assess parents’ adherence, knowledge and attitudes on childhood vaccination program among the Saudi population	180	National Childhood Immunisation Program	0–2 years	Although parents had good adherence, knowledge and positive attitudes on some aspects related to childhood immunisation, gaps in both studied domains were identified
[17]	To assess parental knowledge and attitudes on childhood immunisation among Saudi parents	731	National Childhood Immunisation Program	0–12 years	The parents had good knowledge and positive attitudes on some aspects related to childhood immunisation. However, gaps in both studied domains were identified
[18]	To demonstrate parents’ attitudes towards the influenza vaccine and to identify possible barriers towards having their children vaccinated against influenza	399	Influenza vaccination	>6 months	Although parents’ knowledge level was poor, parental attitudes towards the seasonal influenza vaccine were generally positive. The majority of parents were aware of the seasonal influenza vaccine; however, adherence to receiving the vaccination for both themselves and their children was low
[19]	To review the perception of parents with asthmatic children towards flu vaccination and its influence on their decision to vaccinate their children in KSA	190	Influenza Vaccination	6 months–15 years	Parents agreed with most of the positive statements of perception towards the vaccine against the flu in asthmatic children, including that non-vaccinated children are more likely to contract the flu virus. The rate of vaccination among their children was, however, low and most of the parents believe that vaccination does not prevent flu virus
[20]	To assess the prevalence of influenza vaccine hesitancy among parents, adult patients and HCWs at King Abdulaziz Medical City, Riyadh	300	Influenza Vaccination	Up to 14 years	Influenza vaccination hesitancy in King Abdulaziz Medical City in Riyadh was low (17%)

**Table 4:** Characteristics of data collections instruments.

R	Data Collection Instrument (DCI)	DCI Reference	DCI Characteristics
[12]	WHO standardised questionnaire	Not Relevant (NR)	Article in press—DCI characteristics are not reported in the abstract
[13]	11-item vaccine hesitancy scale, designed by the SAGE group	[21]	Parents completed 10 dichotomous (yes/no) questions, 11 Likert-type scale (strongly agree, agree, neutral, disagree, or strongly disagree) vaccine hesitancy scale questions and 5 open-ended questions
[14]	28-item semi-structured questionnaire	[14]	Questions were asked at a simple reading level in both Arabic and English. The study instrument had 5 sections. The first examined the child’s personal information such as date of birth, gender and nationality. The second was regarding the biodata of the parents and inquired about their financial status, educational background and health status. The third section assessed the caretaker’s perspective on vaccinations and whether they presumed that they have vaccination delays using the Likert scale. The fourth section concentrated on the child’s physical well-being that could affect his/her vaccination status. The last section focussed on possible reasons for vaccine delays. Additionally, a blank space was provided for the caretaker to give additional reasons.
[15]	18-item structured questionnaire	[15]	The questionnaire had 3 sections. The first section focussed on gender, parents’ ages, education, occupation, the number of children less than 5 years old in family and monthly income. The second section assessed parents’ awareness of the benefits associated with and purpose of vaccination, as well as parents’ confidence in recommending vaccinations to others and sources of information about vaccination and immunisation programs in Saudi Arabia. The third section focussed on current practices in vaccination, including the vaccination status of their children, problems experienced in accessing vaccinations, hospital visits associated with adverse events following vaccination and views about religion and childhood vaccinations that are recommended for children up to 5 years according to the immunisation program.
[16]	Arabic validated questionnaire	NR	An Arabic validated questionnaire was used to collect demographic data, education level, time of vaccination, adherence, knowledge about childhood vaccination programs and attitudes of the parents
[17]	The questionnaire was formulated based on questions and answers published by the Ministry of Health, Saudi Arabia	[22]	The questionnaire was thoroughly revised by the research team for validity, comprehensiveness and appropriateness to collect the required information from the targeted population. There were three main sections to collect data on parents’ demographics, parents’ knowledge and attitudes on childhood immunisation. Responses to knowledge questions were recorded as “Yes”, “No” and “Don’t know”. A five-point-Likert scale (“Strongly agree”, “Agree”, “Not sure”, “Disagree” and “Strongly disagree”) was used to assess parents’ attitudes towards childhood immunisation.
[18]	29-question questionnaire	NR	The questionnaire included four sections: Section 1, demographic data; Section 2, influenza vaccine awareness; Section 3, influenza vaccine knowledge; Section 4, attitudes towards the influenza vaccine
[19]	Survey tool adopted from the Triandis model	[23]	The semi-structured questionnaire was developed to obtain data on demographics, knowledge, attitudes, social support, perceived benefits and causes of non-adherence. The respondents’ agreement was measured using the Likert agreement scale
[20]	Authors’ developed and validated questionnaire to evaluate influenza vaccine hesitancy	[20]	The questionnaire included data on demographics (age, sex, chronic illnesses, education level), willingness to take the vaccine both in the past and in the future, reasons for not taking the vaccine, knowledge of the vaccine and sources of this knowledge and confidence in the vaccine

**Figure A2:**
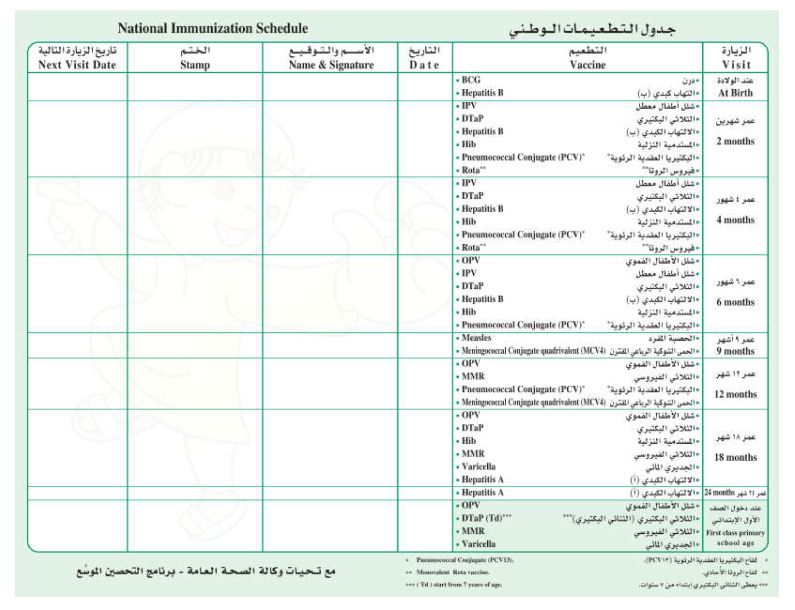
Saudi Vaccination Certificate.

## Appendix A

**Table A1:** Keyword statements and search strategies used for Medline and Embase databases.

Set#	Searched for	Databases
S1	(MESH.EXACT.EXPLODE(“Health Knowledge, Attitudes, Practice”) OR MESH.EXACT.EXPLODE(“Patient Medication Knowledge”) OR MESH.EXACT.EXPLODE(“Knowledge”))	MEDLINE^®^
S2	(MESH.EXACT.EXPLODE(“Attitude”) OR MESH.EXACT.EXPLODE(“Attitude to Health”))	MEDLINE^®^
S3	(ti,ab(“know” or “aware” or “attitude” or “perception” or “health attitude” or “perceive” or “opinion” or “accept” or “belie” or “knowledge” or “intention” or “interest” or “view” or “awareness” or “practice” or “kap” or “KAP”))	MEDLINE^®^
S4	S3 OR S2 OR S1	MEDLINE^®^
S5	(MESH.EXACT.EXPLODE(“Vaccination”) OR MESH.EXACT.EXPLODE(“Immunization”) OR (ti,ab(“vaccin” or “immun”)))	MEDLINE^®^
S6	(MESH.EXACT.EXPLODE(“Child”) OR MESH.EXACT.EXPLODE(“Infant”) OR MESH.EXACT.EXPLODE(“Adolescent”) OR (ti,ab(“boy” or “girl” or “child” or “infant” or “adolescent”)))	MEDLINE^®^
S7	S6 AND S5	MEDLINE^®^
S8	(ti,ab(“saudi arabia”) or ti,ab(“Kingdom of Saudi Arabia”) or ti,ab(“saudi”) or ti,ab(“arabia”))	MEDLINE^®^
S9	S8 AND S7 AND S4	MEDLINE^®^
S10	(S9) and (pd(>20090925)) and (human(yes)) and (la.exact(“English”))	MEDLINE^®^
S11	(S9) and (pd(>20100925)) and (human(yes)) and (la.exact(“English”))	MEDLINE^®^
S12	EMB.EXACT.EXPLODE(“knowledge”) OR (EMB.EXACT.EXPLODE(“attitude to health”) OR EMB.EXACT.EXPLODE(“attitude”)) OR (EMB.EXACT(“perception”) OR EMB.EXACT.EXPLODE(“perception deafness”))	Embase^®^
S13	((ti,ab(“know” or “aware” or “attitude” or “perception” or “health attitude” or “perceive” or “opinion” or “accept” or “belie” or “knowledge” or “intention” or “interest” or “view*” or “awareness” or “practice” or “kap” or “KAP”)))	Embase^®^
S14	S13 OR S12	Embase^®^
S15	EMB.EXACT.EXPLODE(“vaccination”) OR ((ti,ab(“vaccin” or “immun” or “immunisation” or “immunization”)))	Embase^®^
S16	EMB.EXACT.EXPLODE(“adolescent”) OR (EMB.EXACT.EXPLODE(“juvenile”) OR EMB.EXACT.EXPLODE(“child”)) OR EMB.EXACT.EXPLODE(“boy”) OR EMB.EXACT.EXPLODE(“girl”) OR ((ti,ab(“boy” or “girl*” or “child” or “infant” or “adolescent”)))	Embase^®^
S17	S16 AND S15	Embase^®^
S18	((ti,ab(“saudi arabia”) or ti,ab(“Kingdom of Saudi Arabia”) or ti,ab(“saudi”) or ti,ab(“arabia”)))	Embase^®^
S19	S18 AND S17 AND S14	Embase^®^
S20	(S19) and (ud(>20100925)) and (human(yes)) and (la.exact(“English”))	Embase^®^
